# Heat-Killed *Enterococcus faecalis* Inhibit FL83B Hepatic Lipid Accumulation and High Fat Diet-Induced Fatty Liver Damage in Rats by Activating Lipolysis through the Regulation the AMPK Signaling Pathway

**DOI:** 10.3390/ijms24054486

**Published:** 2023-02-24

**Authors:** Jin-Ho Lee, Keun-Jung Woo, Joonpyo Hong, Kwon-Il Han, Han Sung Kim, Tack-Joong Kim

**Affiliations:** 1 Division of Biological Science and Technology, Yonsei University, Wonju 26493, Republic of Korea; 2 Research & Development Center, Bereum Co., Ltd., Wonju 26361, Republic of Korea; 3 Department of Biomedical Engineering, Yonsei University, Wonju 26493, Republic of Korea; 4 Research & Development Center, Doctor TJ Co., Ltd., Wonju 26493, Republic of Korea

**Keywords:** *Enterococcus faecalis*, EF-2001, lipid metabolism, high-fat diet, AMPK signaling

## Abstract

Continuous consumption of high-calorie meals causes lipid accumulation in the liver and liver damage, leading to non-alcoholic fatty liver disease (NAFLD). A case study of the hepatic lipid accumulation model is needed to identify the mechanisms underlying lipid metabolism in the liver. In this study, the prevention mechanism of lipid accumulation in the liver of *Enterococcus faecalis* 2001 (EF-2001) was extended using FL83B cells (FL83Bs) and high-fat diet (HFD)-induced hepatic steatosis. EF-2001 treatment inhibited the oleic acid (OA) lipid accumulation in FL83B liver cells. Furthermore, we performed lipid reduction analysis to confirm the underlying mechanism of lipolysis. The results showed that EF-2001 downregulated proteins and upregulated AMP-activated protein kinase (AMPK) phosphorylation in the sterol regulatory element-binding protein 1c (SREBP-1c) and AMPK signaling pathways, respectively. The effect of EF-2001 on OA-induced hepatic lipid accumulation in FL83Bs enhanced the phosphorylation of acetyl-CoA carboxylase and reduced the levels of lipid accumulation proteins SREBP-1c and fatty acid synthase. EF-2001 treatment increased the levels of adipose triglyceride lipase and monoacylglycerol during lipase enzyme activation, which, when increased, contributed to increased liver lipolysis. In conclusion, EF-2001 inhibits OA-induced FL83B hepatic lipid accumulation and HFD-induced hepatic steatosis in rats through the AMPK signaling pathway.

## 1. Introduction

As the percentage of high-calorie diets increases and the diet composition of modern society changes [1], overweight and obesity population rates are increasing not only in Korea but also globally [2,3]. Continuous intake of a high-fat diet induces lipid accumulation rather than energy consumption in the body [4]. Excess energy is stored in the form of body fat, which eventually affects lipid metabolism [5]. Regulation of lipid metabolism homeostasis is essential for maintaining the lipid balance in the body [6]. Diabetes, obesity, fatty liver disease, and cardiovascular disease are caused by impaired lipid metabolism [7]. As a result, current research is primarily focused on studying the mechanism underlying lipid metabolism to effectively prevent and improve associated disorders [8,9].

Non-alcoholic fatty liver disease (NAFLD) is associated with obesity and type 2 diabetes dyslipidemia and is used to determine the degree of metabolic syndrome in the liver [10,11]. The lipid accumulation model of liver cells induced with oleic acid (OA) is widely used to obtain baseline data for fatty liver research, and includes FL83B, HepG2, and huH7 cells [12,13,14,15]. OA-induced intracellular lipid accumulation proceeds via the activation of lipid synthesis pathways, such as the sterol regulatory element-binding protein 1c (SREBP-1c) and peroxisome proliferator-activated receptor (PPAR)-γ pathways [16,17]. It also reduces lipolysis processes, such as the activity of AMP-activated protein kinase (AMPK) and lipase. In particular, studies on lipid accumulation in the liver through OA induction in FL83B cells (FL83Bs) and control of the lipid decomposition pathway have primarily focused on the AMPK signaling pathway and lipase enzyme activity [18,19].

AMPK is a vital sensor that uses AMP to generate energy when the energy in the body is depleted. It plays an important role in lipid and carbohydrate metabolism in the liver and is a major factor in the recovery from obesity and diabetes [20,21,22]. In contrast, AMPK decreases lipid accumulation by regulating PPAR-α expression [23].

AMPK activity can induce acetyl-CoA carboxylase (ACC) phosphorylation and decrease ACC activity to suppress lipid biosynthesis [24]. Thus, phosphorylation of AMPK not only maintains energy balance but also inhibits the formation of triglycerides (TGs) to reduce lipid accumulation in the liver. In addition, sirt 1 plays a role in regulating AMPK activity to enhance AMPK phosphorylation in adipocytes and hepatocytes [25].

Hepatic lipid synthesis is performed by the transcription and translation of genes including *SREBP-1* and fatty acid synthase (*FAS*) [26,27]. Hepatocytes activate adipose triglyceride lipase (ATGL), hormone-sensitive lipase (HSL), and monoacylglycerol (MGL) to decompose TGs and form glycerol and free fatty acids during the citric acid cycle for energy production [28,29]. Decomposed free fatty acids stimulate macrophages in the liver to cause an inflammatory reaction and activated macrophages release inflammatory mediators to induce insulin resistance in liver cells [30,31].

*Enterococcus faecalis* promotes intestinal microbiota balance, alleviates metabolic syndrome, and modulates immunity, among other functions [32]. *E. faecalis* is also effective in treating hyperlipidemia, obesity, and fatty liver disease [33]. Probiotic strains of *E. faecalis* have been identified though isolation from fecal samples of healthy individuals [34]. It has been demonstrated that *E. faecalis* is not only beneficial when alive but is also beneficial when dead [35]. Recently, the genome sequence of EF-2001 was revealed, and it was found to significantly inhibit depression by enhancing pre-frontal local myelination [36]. EF-2001 has been shown to have beneficial effects on human health. These include radioprotective, antitumor, anti-inflammatory, anti-atopic dermatitis, and muscle atrophy prevention [37,38,39,40]. In animal models of prostatic hyperplasia, EF-2001 was also found to be effective [41]. Previous studies have reported that certain products utilizing bacteria, such as *Lactobacillus plantarum* NCU116, *Lactobacillus acidophilus* NX2-6, and other *Lactobacillus* strains that overexpress bile salt hydrolase, can inhibit hepatic accumulation of lipids [42]. Several other studies have reported the inhibitory effects of bacterial products on lipid accumulation, including products utilizing bacteria, such as *Lactobacillus sakei* ADM14, *L. brevis* OPK-3, and *L. plantarum* LMT1-48 [43,44,45,46]. Recently, we demonstrated that administrating the EF-2001 exhibits an anti-obesity effect in high-fat diet (HFD)-induced rats. Our results showed that the intake of EF-2001 significantly prevented HFD-induced obesity in rats by inhibiting the C/EBP-α and PPAR-γ in the insulin signaling pathway, thus reducing lipid accumulation [47]. Another study reported that heat treatment of *E. *faecalis** FK-23 could ameliorate HFD-induced obesity in mice. The inhibitory effect of FK-23 on hepatic steatosis in HFD-fed mice was induced by the prevention of fat accumulation in the liver through modulation of the activities of genes involved in hepatic fatty acid oxidation [48]. Mishra and Ghosh reported on the synergistic effect of the probiotic *E. faecalis AG5* on HFD-induced obesity and the role of propionic acid (PA) in the induction of apoptosis in 3T3-L1 pre-adipocytes [33]. AG5 was found to reduce adipocyte hypertrophy and fatty acid accumulation. This study revealed low PPARγ activity inhibiting 5-LOX, which may be related to adipose apoptosis, and that 5-LOX inhibition increased caspase activity. This is associated with the initiation of cell death [33]. Fan et al. reported that heat-killed *E. faecalis* improved the abnormal hepatic lipid mechanism in diet-induced obese (DIO) mice by reducing triglyceride (TG) accumulation [49]. This suggests that administrating the EF-2001 may be effective in attenuating hepatic steatosis, as atherogenic dyslipidaemia has been found to be associated with hepatic steatosis, after adjusting for obesity, physical activity, and hyperglycemia [50].

In this study, the effect of heat-killed *E. faecalis*, EF-2001 on liver lipid accumulation in HFD-induced rats was investigated, and the effects of lipase enzyme activity and AMPK signaling pathways were investigated to provide a new theoretical basis for the treatment of liver lipid metabolic disorders.

## 2. Results

### 2.1. EF-2001 Intake Effectively Prevents Fatty Liver Tissue and Liver Damage in HFD-Induced Rats

**Figure 1:**
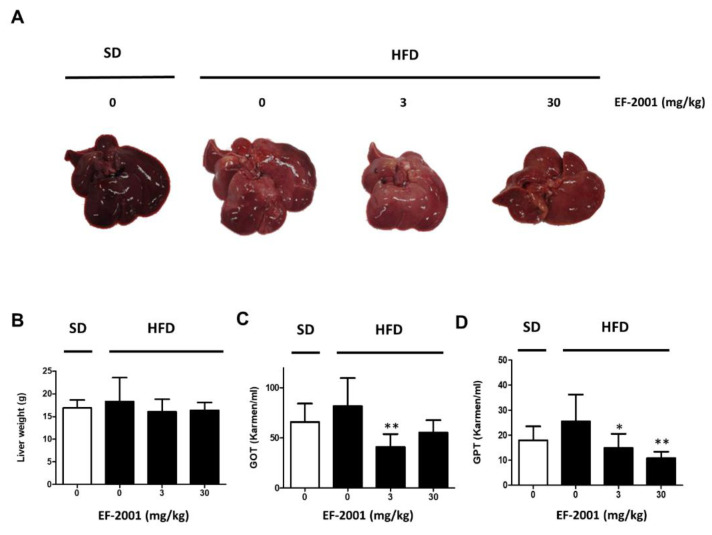
Effects of EF-2001 oral administration on high fat diet (HFD)-induced accumulation of liver tissue and level of glutamic oxaloacetic transaminase/glutamic pyruvic transaminase (GOT/GPT) in serum of obese rats. Male rats were divided into standard diet (SD) or HFD groups and were orally administered refined water or 3 mg/kg or 30 mg/kg EF-2001 in water (n = 6 rats/group). Gavage was continued for 6 weeks. After six weeks of feeding, rats were fasted for 12 h, and then, blood samples were collected. (**A**) Effects of EF-2001 on the accumulation of liver tissue of HFD-induced hepatic steatosis. Pictures were taken for livers of each group. (**B**) Effects of EF-2001 on the liver weight of rats fed SD and HFD for 6 weeks. To investigate the weight of liver tissue, the rats were sacrificed, and liver tissue weight was measured. (**C**) Effects of EF-2001 on GOT in HFD-induced hepatic steatosis. (**D**) Effects of EF-2001 on GPT in HFD-induced hepatic steatosis. Data was analyzed with liver tissue weight per body weight. Data are represented as the mean ± SEM. (n = 6); * p < 0.05 and ** *p* < 0.01 vs. HFD-fed rats alone without EF-2001.

To establish HFD-induced hepatic steatosis, male rats were divided into SD or HFD groups. Rats were orally administered refined water or EF-2001 in water at each dose per day, as scheduled. HFD groups were subcategorized into three groups (only refined water, 3 mg/kg, or 30 mg/kg EF-2001 in water) to evaluate the effects of EF-2001 on fatty liver-induced rats. We investigated the effects of EF-2001 intake on HFD-induced elevation in non-alcoholic fatty liver disease (NAFLD). Rats fed the HFD weighed significantly higher than rats fed the SD. In the HFD group, brightened and enlarged livers with fat accumulation were observed. Among the HFD groups, the appearance of the liver with accumulated bright-toned fat in the EF-2001 group rats was similar to that of the HFD group (Figure 1A), but the size of the liver was similar to that of the SD group rats. Both groups of HFD rats were administered 3 mg/kg or 30 mg/kg EF-2001 and demonstrated a reduction in liver weight (Figure 1B). 

Both glutamic oxaloacetic transaminase (GOT) and glutamic pyruvic transaminase (GPT) levels were significantly increased by the HFD. EF-2001 recovered GOT and GPT levels in both the 3 mg/kg and 30 mg/kg groups, and the levels of GOT and GPT were significantly reduced in both EF-2001 administration groups due to liver damage caused by high-fat diet induction (Figure 1C,D). These results show that EF-2001 intake downregulated HFD-induced fatty liver damage.

### 2.2. Effect of EF-2001 on Oleic Acid-Induced Hepatic Lipid Accumulation in FL83Bs

**Figure 2:**
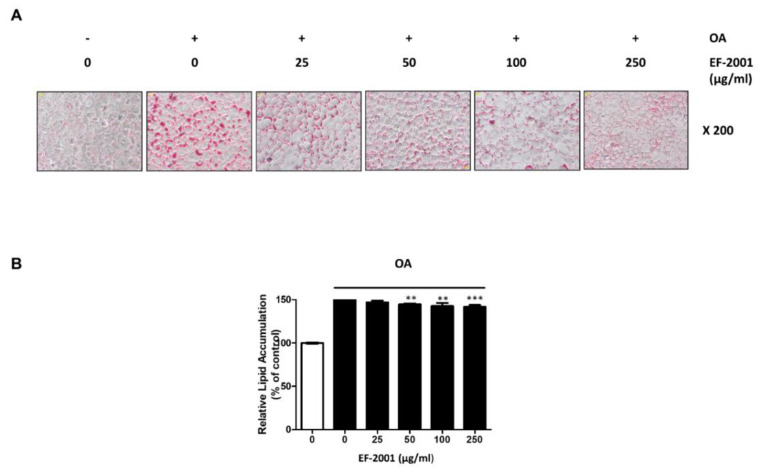
Effects of EF-2001 on lipid accumulation in FL83B hepatocytes. (**A**) Effects of EF-2001 on lipid accumulation induced FL83B hepatocytes with ORO staining. FL83B hepatocyte lipid accumulation induced by 0.5 mM oleic acid (OA) for 48 h. FL83B hepatocyte media was changed for new serum free media in the absence or presence of EF-2001 (25–250 μg/mL) at 24 h. Lipid accumulation of FL83B hepatocyte was observed by ORO staining. (**B**) Effects of EF-2001 on the relative lipid accumulation of FL83B hepatocyte. ORO stained FL83Bs were eluted with 100% isopropanol, and optical density (O.D.) was measured at 490 nm. Data are represented as mean ± SEM. (*n* = 4); ** *p* < 0.01, and *** *p* < 0.001 vs. control.

We measured the hepatic lipid accumulation with or without EF-2001 in FL83Bs to investigate how EF-2001 contributes to lipid accumulation in oleic acid (OA)-induced FL83Bs. The effects of EF-2001 on OA-induced hepatic lipid accumulation in FL83Bs were examined by ORO staining. FL83Bs were pretreated with OA (0.5 mM) in serum-free medium for 48 h and then treated with EF-2001 (0, 25, 50, 100 or 250 μg/mL) for 24 h. OA-induced cells showed significantly increased lipid accumulation in the FL83Bs. However, treatment with EF-2001 (0, 25, 50, 100 or 250 μg/mL) significantly decreased OA-induced lipid accumulation in FL83Bs (Figure 2).

### 2.3. Effect of EF-2001 on Neutral Lipid Droplet of Oleic Acid-Induced FL83Bs Hepatic Lipid Accumulation

**Figure 3:**
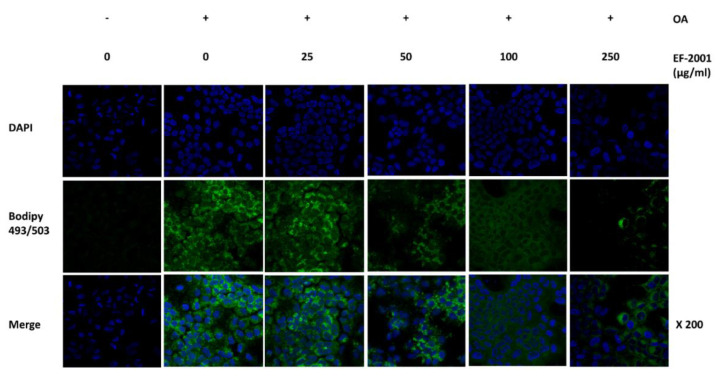
Effect of EF-2001 on confocal microscopy detection of neutral lipid droplet by BODIPY 493/507 in FL83B hepatocyte. FL83B hepatocyte lipid accumulation induced by 0.5 mM oleic acid (OA) for 48 h. FL83B hepatocyte media was changed in the presence of various doses of EF-2001 (0, 25, 50, 100, and 250 μg/mL) for 24 h. Neutral lipid was marked by BODIPY 493/503 (green), and nuclei were stained with 4′,6-diamidino-2-phenylindole (blue). The stained neutral lipid was represented with confocal microscopy at ×200 magnification.

We conducted confocal microscopy in OA-induced FL83Bs to investigate how lipogenesis and lipolysis occur during lipid accumulation in EF-2001 (0, 25, 50, 100, or 250 μg/mL). After 48 h of OA induction, lipid synthesis increased in the control group. In the EF-2001-treated group, intracellular neutral lipid droplets decreased for up to 24 h (Figure 3). Therefore, we confirmed that EF-2001 inhibited intracellular lipid accumulation.

### 2.4. Effects of EF-2001 on the Expression of Lipase Enzyme Protein in FL83Bs

**Figure 4:**
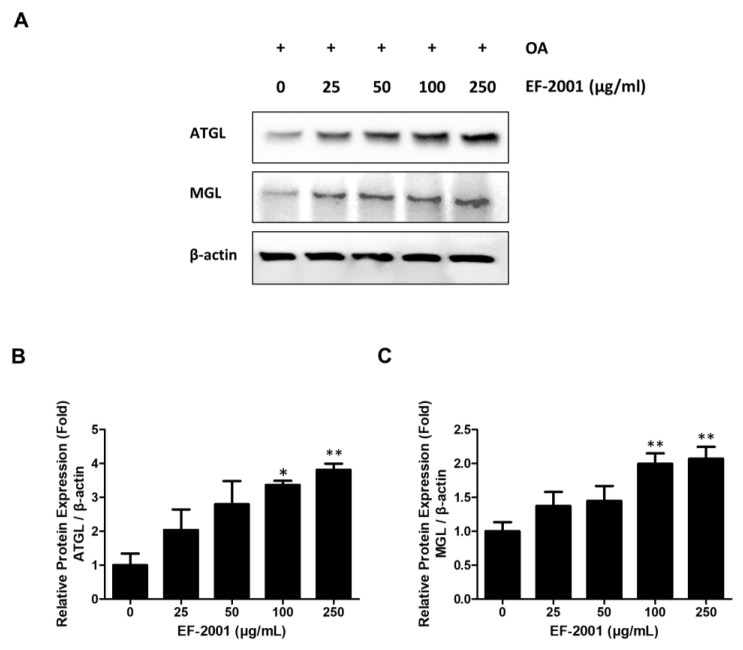
Effects of EF-2001 on lipase in FL83B hepatocytes. FL83B hepatocytes were treated with 0.5 mM oleic acid (OA) for 48 h to induce lipid accumulation. Media was changed in the absence or presence of various doses of EF-2001. Cells were harvested at the time at which the expression of each lipase protein was induced, and Western blotting was performed to determine the expression levels of (**A**). (**B**) Cells were sampled by lysis buffer after EF-2001 treatment for adipose triglyceride lipase (ATGL) (6 h). (**C**) Cells were sampled after EF-2001 treatment for monoacylglycerol (MGL) (12 h). Data are represented as mean ± SEM. (*n* = 3); * *p* < 0.05 and ** *p* < 0.01 vs. control.

After OA induction, ATGL and MGL expressions were observed within 24 h of EF-2001 treatment in FL83Bs. ATGL, an early lipolytic enzyme protein, showed increased expression in EF-2001-treated FL83Bs in a dose-dependent manner. EF-2001 also increased the expression of MGL, a late signal of lipolysis, in a dose-dependent manner in FL83Bs (Figure 4). Additionally, it was found to EF-2001 contributed to lipase activation in OA-induced hepatic lipid accumulation in FL83Bs.

### 2.5. Effects of EF-2001 on the Expression of AMPK and SREBP Signaling Pathway

**Figure 5:**
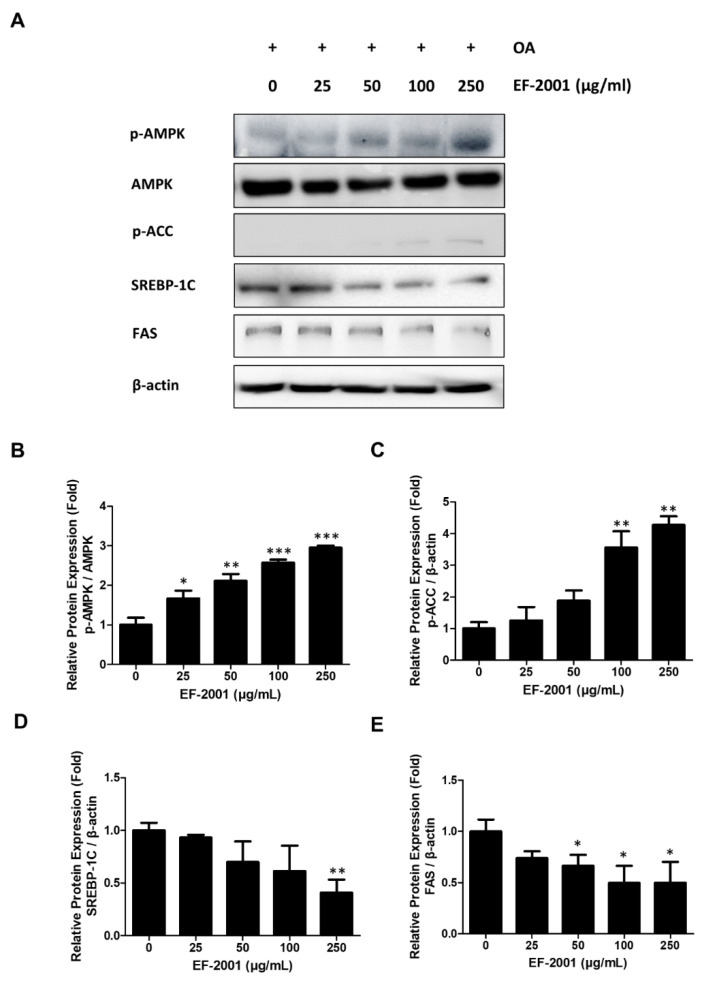
Effects of EF-2001 on AMP-activated protein kinase (AMPK) signaling pathways in FL83B hepatocytes. FL83B hepatocytes were treated with 0.5 mM oleic acid (OA) for 48 h to induce lipid accumulation. Media was changed in the absence or presence of various doses of EF-2001. Cells were harvested at the time at which the expression of each AMPK signaling protein was induced, and Western blotting was performed to determine the expression levels of (**A**). The relative protein expression levels of p-AMPK (**B**), p-ACC (**C**), SREBP-1C (**D**), and FAS (**E**) are shown in parts, respectively. Data are represented as mean ± SEM. (*n* = 3); * *p* < 0.05, ** *p* < 0.01, and *** *p* < 0.001 vs. control.

To identify the mechanism of lipolysis induced by EF-2001, the effects of EF-2001 on AMPK and SREBP signaling pathways were investigated. We compared AMPK signaling pathway-related proteins (AMPK and ACC) and lipid synthesis-related proteins (SREBP-1C and FAS) in OA-induced FL83B hepatocytes. The treatment of EF-2001 significantly increased the expression of phosphorylated AMPK and ACC in a dose-dependent manner (Figure 5B,C). EF-2001 treatment decreased SREBP-1C and FAS expressions in a dose-dependent manner (Figure 5D,E). Thus, these results showed that EF-2001 treatment of hepatic lipid decomposition in lipogenesis of OA-induced FL83Bs was due to activation of the AMPK signaling pathway.

### 2.6. Effects of EF-2001 on AMPK Targeted Signaling Pathway

**Figure 6:**
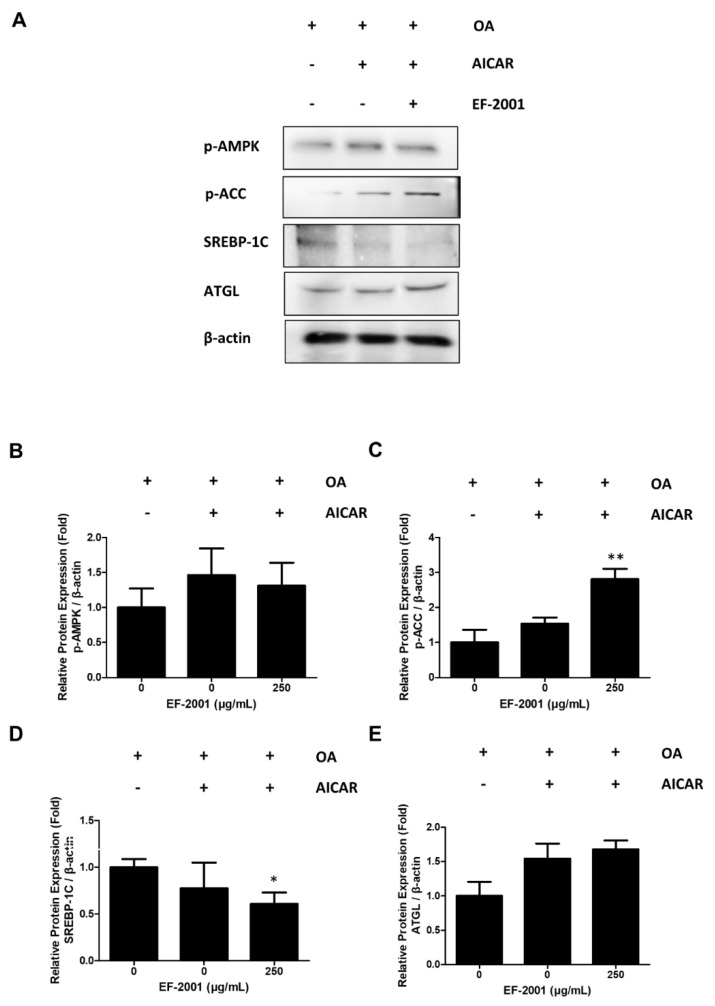
Effects of EF-2001 on AMPK signaling pathways with 5-aminoimidazole-4-carboxamide-1-β-D-ribofuranoside (AICAR) and compound C in FL83B hepatocytes. The AMPK signaling pathway proteins were detected by Western blot. (**A**) FL83Bs were treated with 0.5 mM oleic acid (OA) for 48 h, followed by EF-2001 (250 μg/mL) with or without AMPK enhancer (AICAR) for 24 h. The relative protein expression levels of p-AMPK (**B**), p-ACC (**C**), SREBP-1C (**D**), and ATGL (**E**) are shown in the graphs. The fold expression levels were measured relative to the expression of β-actin. Three independent experiments were analyzed, and the fold expression levels were measured relative to the expression of β-actin. Data are presented as mean ± SEM; * *p* < 0.05 and ** *p* < 0.05 compared with OA group.

**Figure 7:**
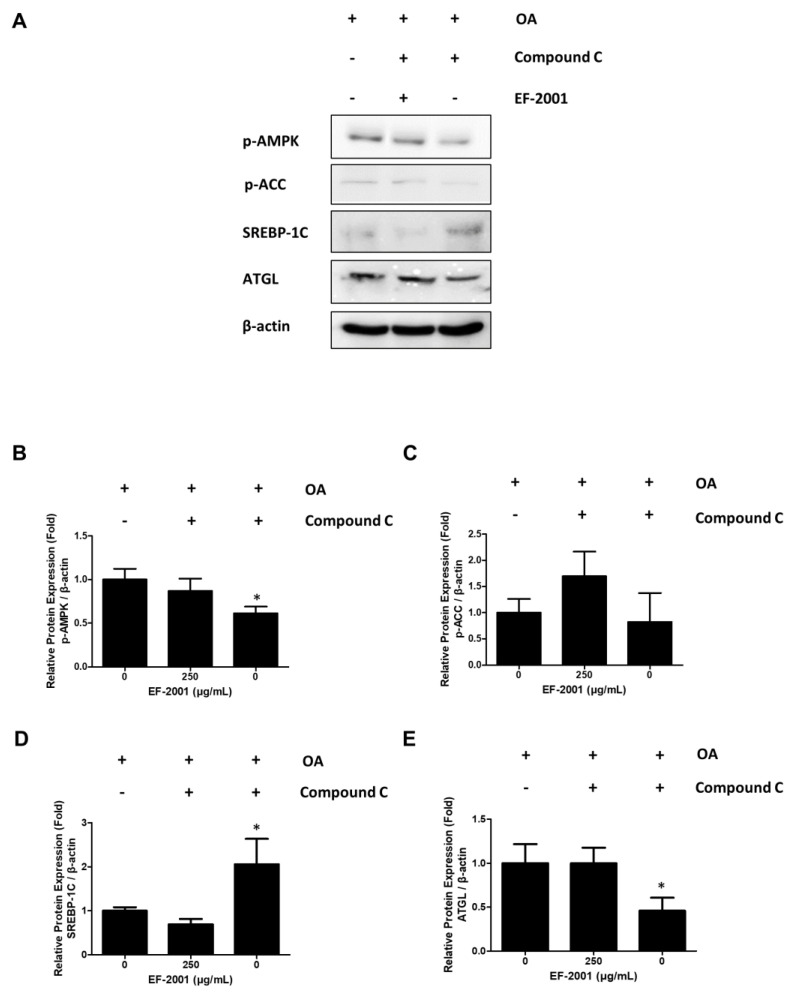
Effects of EF-2001 on AMPK signaling pathways with AICAR and compound C in FL83B hepatocytes. The AMPK signaling pathway proteins were detected by western blotting analysis. (**A**) FL83Bs were treated with 0.5 mM oleic acid (OA) for 48 h, followed by EF-2001 (250 μg/mL) with or without AMPK inhibitor (compound C) for 24 h. The relative protein expression levels of p-AMPK **(B**), p-ACC (**C**), SREBP-1C (**D**), and ATGL (**E**) are shown in the graphs. The fold expression levels were measured relative to the expression of β-actin. Three independent experiments were analyzed, and the fold expression levels were measured relative to the expression of β-actin. Data are presented as mean ± SEM; * *p* < 0.05 compared with the OA group.

We conducted experiments to determine the relationship between the expression of lipid-related biomarkers and treatment with EF-2001 due to AMPK signal changes using activators (AICAR) and inhibitors (compound C) of AMPK as AMPK targets. In addition, we observed that AMPK phosphorylation was increased in FL83Bs treated with AICAR. However, FL83Bs treated with AICAR and EF-2001 showed increased ACC phosphorylation and ATGL expression (Figure 6). Interestingly, co-treatment of the AMPK inhibitors, compound C and EF-2001, with FL83Bs resulted in the phosphorylation of AMPK and ACC and the expression of a lipase protein, ATGL. We observed a significant difference in the degree of inhibition of SREBP-1C expression in the EF-2001-treated group (Figure 7).

### 2.7. Effects of EF-2001 AMPK Signaling Pathway on HFD Induced Fatty Liver

**Figure 8:**
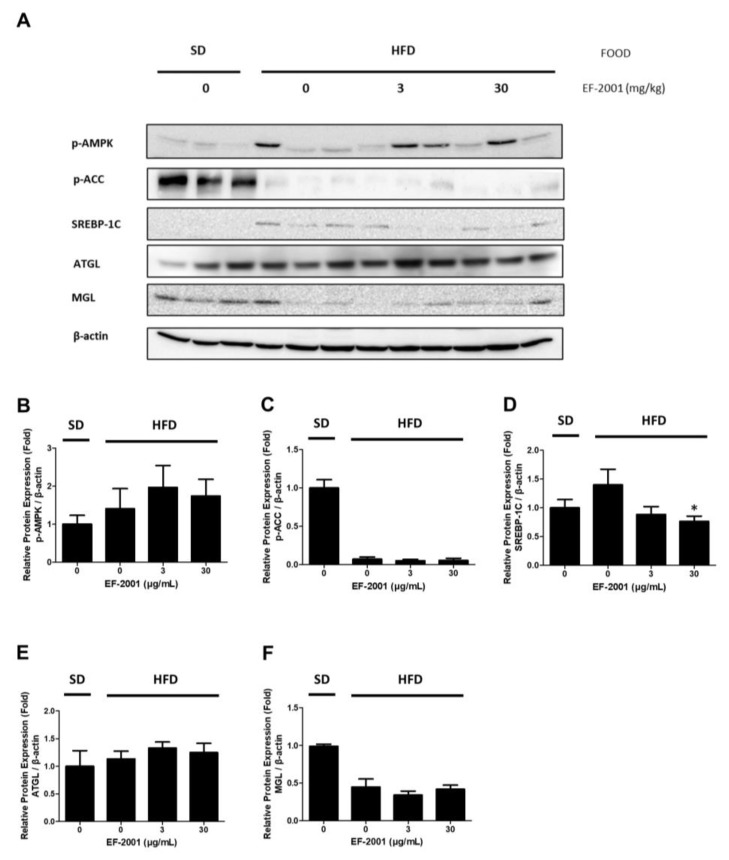
Effects of EF-2001 on lipase and AMPK signaling pathways in HFD-induced fatty liver. Male rats were divided into SD or HFD groups and orally administered refined water, 3 mg/kg or 30 mg/kg EF-2001 for 6 weeks (*n* = 6 rats/group). Lipase and AMPK signaling pathway were detected by immunoblotting. (**A**) The protein levels of p-AMPK, SREBP-1C, p-ACC, ATGL, and MGL were standardized depending to the amount of β-actin. The relative protein expression levels of p-AMPK (**B**), SREBP-1C (**C**), p-ACC (**D**), ATGL (**E**), and MGL (**F**) are shown in the graphs. Data are represented as the mean ± SEM. (*n* = 6); * *p* < 0.05 vs. HFD-fed rats alone without EF-2001.

Finally, we investigated the effect of EF-2001 on the protein expression level of the AMPK signaling pathway in the liver tissues of SD- and HFD-fed rats. During hepatic lipid accumulation, p-AMPK was up-regulated in the EF-2001 group. In addition, experiments were conducted during lipogenesis to investigate how EF-2001 treatment **of** the expression levels of lipolysis-related proteins such as p-AMPK, p-ACC, and ATGL (Figure 8A). In the HFD group, AMPK phosphorylation was increased by EF-2001 treatment (Figure 8B). However, ACC phosphorylation did not changed (Figure 8C). Moreover, oral administration of EF-2001 (30 mg/kg) to the HFD group effectively decreased the protein expression level of SREBP-1C to a level lower than that in the untreated EF-2001 HFD group (Figure 8D). In addition, ATGL protein expression increased in the 3 mg/kg and 30 mg/kg EF-2001-treated groups (Figure 8E). However, there was no significant difference in the MGL expression in the HFD group (Figure 8F).

## 3. Discussion

In our previous studies, we reported the effect of downregulation of total cholesterol (such as TG and low-density lipoprotein (LDL)-cholesterol levels), leading to an increased potential of NAFLD in HFD-induced rats by EF-2001 [47]. Downregulation of LDL levels may be an important strategy for the prevention of NAFLD. Since oral administration of EF-2001 suppressed LDL levels, we hypothesized that EF-2001 would have an effect on NAFLD. Therefore, follow-up experiments were conducted to determine the effects and molecular mechanisms underlying NAFLD.

We demonstrated that the oral administration of EF-2001 lowered GOT and GPT levels at both doses (3 mg/kg and 30 mg/kg EF-2001). In addition, both doses reduced the liver weight in HFD-fed rats (Figure 1). Our results indicate that EF-2001 administration decreases liver damage by reducing the physical size of the HFD-induced fatty liver and lowering the levels of enzymes, such as GOT and GPT, released in the blood following liver damage. 

FL83Bs are a normal component of liver tissue, and lipid accumulation in FL83Bs plays an important role in the mechanism of lipogenesis, lipolysis, and the onset of NAFLD [51]. We examined the effects of EF-2001 on lipid accumulation in FL83B hepatocytes stained with ORO, and confirmed that treatment with EF-2001 inhibited hepatic lipid accumulation (Figure 2). Furthermore, we observed a reduction in lipid droplets following EF-2001 treatment in FL83B hepatocytes (Figure 3).

To confirm the anti-lipid accumulation effect of EF-2001, we analyzed the molecular mechanisms by which EF-2001 inhibits lipid accumulation in FL83Bs. Several studies have noted the importance of lipolytic enzymes such as ATGL and MGL in the regulation of hepatic lipid accumulation [52,53]. Our results also showed that ATGL and MGL levels increased in a dose-dependent manner upon EF-2001 treatment, proving that EF-2001 contributes to an increase in lipolytic enzyme expression in FL83B hepatocytes (Figure 4).

Hence, we hypothesized that EF-2001 could reduce lipid synthesis and increase lipolysis by activating the AMPK pathway to inhibit the development of fatty liver cells. EF-2001 inhibited the expression of SREBP-1C signaling pathway unit proteins, such as SREBP-1C and FAS, which mediate the lipid accumulation process (Figure 5). We found that EF-2001 can significantly enhance AMPK phosphorylation but can also enhance ACC phosphorylation to inhibit the synthesis of fatty acid chains (Figure 5).

AMPK is an energy regulator that assists in regulating glucose and lipid metabolism to maintain the cellular energy balance [54]. Recent studies have also reported a relationship between the AMPK signaling pathway and lipolysis [55]. AMPK activation induces lipolysis in hepatocytes. It also plays a crucial role in lipolysis progression [56,57]. The SREBP-1C pathway is downregulated by phosphorylated AMPK [55]. ACC-1 is responsible for synthesizing fatty acids and can be controlled by inhibitory phosphorylation by AMPK [58]. Confirming the relationship between AMPK and EF-2001, recovered or increased ACC and AMPK phosphorylation in hepatocytes treated with compound C, an AMPK inhibitor, or AICAR, an AMPK activator (Figure 6 and Figure 7). This is related to the inhibition of the AMPK signaling pathway, which is a key factor in lipogenesis. Consequently, EF-2001 induced the phosphorylation of AMPK and many kinds of lipases and inhibited the expression of the SREBP-1C signaling pathway in HFD-induced obese rats (Figure 8). Therefore, we suggest that EF-2001 inhibits fatty liver cell development, and it can cause the inhibition of NAFLD through AMPK phosphorylation.

In addition to the metabolites produced by specific members of the microbial community, there are also metabolites that are consumed or transformed by bacteria outside the microbial community, making gut microbial metabolism a highly complex process. Although the composition of the microbial community determines the metabolism of microorganisms in the intestine, the substrates available to the microbial community is the most important factor since the metabolites produced from specific substrates reflect gut microbial metabolism [59]. 

When food is consumed, certain components containing choline groups in the food are metabolized by GM in the intestine and produce GM-derived products such as trimethylamine (TMA), short-chain fatty acids (SCFAs), and trimethylamine N-oxide (TMAO). Metabolites, such as TMA and TMAO, have been identified as the causative agents of metabolic diseases in animal models and human clinical studies. Recent research has revealed that GM and its metabolites play an important role in the development and progression of cardiovascular and metabolic diseases [60,61,62,63,64]. Therefore, in the process of developing drugs for obesity and metabolic diseases, the metabolic pathways in which these GM-derived metabolites are synthesized can be considered as more important as they can become new therapeutic target sites. Currently, our results do not identify the bacteria capable of modulating the host TMA/TMAO and this aspect needs to be analyzed in future studies.

In some studies, the relationship between *Enterococcus* and liver injury has been described. Ray et al. reported that the ratio of *E. faecalis* associated with cytolysin was increased in the feces of patients with alcoholic fatty liver, while Lang et al. reported that the ratio of *E. faecalis* associated with cytolysin increased in non-alcoholic fatty liver patients, although this finding is under debate [65,66]. In a study by Tan, nonalcoholic fatty liver was inhibited by reducing *Roseburia, Intestinibacter,* and *Enterococcus* [67]. There are various other claims; therefore, more research is required. Since the gut microbiome changes dramatically even with a high-fat diet, we are currently conducting a clinical trial to analyze the effects of fecal *E. faecalis* on obesity and metabolic diseases. 

Intestinal microbes have been implicated in the pathogenesis of gastrointestinal diseases and metabolic syndromes, such as obesity. Microbes can also produce metabolites, genetic products, and exhibit pathogenic potential that can negatively affect the host [68]. Therefore, it is important to investigate the potential negative effects of consuming heat-treated dead cells of *Enterococcus* in future studies.

In order to calculate the balance of benefits and harms caused by the gut microbiome from the host’s perspective, a comprehensive analysis of the distribution, diversity, species composition, and metabolites of the microbiome must be performed. For example, the production of SCFA and vitamins by the gut microbiome has positive effects on energy supply and nutrition, but if this process is out of the normal range, it can lead to disease. Although there are no studies on *E. faecalis* in the intestine with a high-fat diet, we are currently conducting clinical trials, and we plan to analyze the distribution of *E. faecalis* in the feces, changes in structural function, and altered function.

We recently discovered that *E. faecalis* EF-2001 inhibits toll-like receptor (TLR) signaling via anti-inflammatory effects [39]. This suggests the possibility of suppressing metabolic syndromes such as obesity and NAFLD. When the composition of the gut microbiome changes, the uptake of TLR4 ligands (e.g., LPS) and TLR9 ligands (e.g., bacterial DNA) is increased and delivered to the liver through the portal vein. Therefore, blocking TLR signaling in the liver could suppress the expression of metabolic syndromes such as obesity, NAFLD, and NASH [69]. Microbial-derived SCFA (acetate, butyrate, and propionate) may be beneficial to the host as sources of carbon and energy. In fact, many studies on the beneficial effects of SCFA on obesity, appetite, and inflammation in the colon have been published [70,71,72]. The structure of heat killed *E. faecalis* or the secreted substances that modulate the activity of the lipid pathway were not identified in this study. However, these findings provide a starting point for further research on the potential association between heat-killed *E. faecalis* and metabolic diseases such as obesity and fatty liver. 

We concluded that EF-2001 directly lowered hepatic lipid accumulation through the regulation of AMPK signaling. In conclusion, our study suggests that EF-2001 may be a promising candidate for reducing various diseases, including liver damage in obese individuals, by decreasing liver lipid accumulation. However, further research is needed to identify the specific components of EF-2001 that modulate lipogenesis and lipolysis mechanisms to gain a better understanding of its potential therapeutic effects.

As next-generation sequencing became common after the 2000s, metagenomic research has become increasingly popular. This analysis method has shown that various factors, such as diet, race, age, antibiotics, stress, psychological factors, maternal health, birth methods such as natural childbirth, environmental factors, and exercise affect the distribution of intestinal microbes [73]. However, research on each of these factors is still incomplete, and more studies are needed to fully understand their impact on the gut microbiome.

## 4. Materials and Methods

### 4.1. Preparation of Heat-Killed Enterococcus faecalis (EF-2001)

EF-2001, originating from human feces, is a merchantable parabiotic purified from Bereum Co., Ltd. (Wonju, Republic of Korea) and supplied as a heat-killed, dried powder. Prior to being heat-killed, dried EF-2001 contained 7.5 × 10^12^ units per gram.

### 4.2. Chemical Reagent

5-aminoimidazole-4-carboxamide-1-β-D-ribofuranoside (AICAR, an AMPK activator) and compound C (an AMPK inhibitor) were purchased from Sigma-Aldrich (St. Louis, MO, USA).

### 4.3. Animal Experiments

Twenty-four male Sprague–Dawley rats (3-week-old) with an initial body weight of 30–40 g were purchased from Orient Bio Tech Laboratories (Gyeonggi, Republic of Korea), and, used in the experiments. The rats were acclimatized for one week, and food intake measurements were initiated. The rats were divided into four groups (*n* = 6/group) according to their diet. For experimental procedures in HFD-induced obese rats, the rats in the standard diet (SD) group were fed a commercial diet (5L79, Lab Diet Inc., St. Louis, MO, USA) and those in the HFD group were fed an HFD diet (D12492, Research Diets, Inc., New Brunswick, NJ, USA) for six weeks. Rats were subcategorized into three groups: water only, 3 mg/kg EF-2001, and 30 mg/kg EF-2001 in water. Water and food were provided ad libitum, all times. Rats in the HFD group were orally administered pure water or EF-2001 (3 or 30 mg/kg) in water once daily. Gavage was continued for 6 weeks. All experimental procedures were approved by the Institutional Animal Care and Use Committee of Yonsei University and were performed in accordance with approved guidelines (YWCI-202102-003-01).

### 4.4. Serological Analysis

Blood serum was sampled at six weeks by heart puncture under ether anesthesia using a sterilized vacutainer tube. Serum samples were analyzed to determine the activities of hepatoenzymes, including alanine aminotransferase (ALT) and aspartate transaminase (AST), using ALT and AST detection kits purchased from Asan Pharmaceutical (Seoul, Republic of Korea). The kits were used in accordance with the manufacturer’s instructions.

### 4.5. Cell Culture and Induced Fatty Liver Cells

FL83Bs, purchased from the American Type Culture Collection, were maintained in F12K medium, supplemented with 10% fetal bovine serum, 1% penicillin, and 1% streptomycin (Sigma-Aldrich). Cells were cultured at 37 °C in an incubator with 5% CO_2_. FL83Bs were seeded in a complete medium for 24 h and then incubated with OA (0.5 mM) for 48 h to induce lipid accumulation. The cells were treated with or without EF-2001 (0, 25, 50, 100 or 250 μg/mL) for 24 h to analyze the experimental results.

### 4.6. Oil Red O Staining of FL83B Hepatocyte 

Lipid accumulation was determined by Oil red O (ORO) staining. EF-2001 was treated with differentiation induction medium at doses of 0, 25, 50, 100, and 250 μg/mL at 100% cell confluence, followed by the induction of lipid accumulation in FL83Bs. FL83Bs were washed with phosphate-buffered saline (PBS), fixed with 3.7% formaldehyde (Junsei Chemical, Tokyo, Japan) diluted in PBS, and stained with 60% ORO diluted in distilled water. Once the stain was eluted with 100% isopropanol, lipid accumulation was quantified at 490 nm wavelength using a microplate reader (Molecular Devices, San Jose, CA, USA). The results are presented in the graphs. The percentage of ORO staining was relative to that of the untreated control cells, representing the percentage of stained intracellular lipid droplets.

### 4.7. Western Blot Analysis

FL83Bs and liver tissues of HFD-induced rats were treated with EF-2001, and each protein was added to lysis buffer (iNtRON Biotechnology Inc., Sungnam, Republic of Korea) at the appropriate stage of hepatic lipid accumulation. After sonication, proteins were quantified and tested using the Bradford assay (Bio-Rad, Hercules, CA, USA) for Western blotting. The sodium dodecyl sulfate–polyacrylamide gel electrophoresis ratio was determined based on the molecular weight of the protein. Electrophoresis was performed at 100 V for approximately 2 h. Antibody treatment was performed with primary antibodies (SREBP-1C, P-AMPK, AMPK, FAS, P-ACC, ACC, ATGL, P-HSL, MGL, CD36, and β-actin) at a rate of 1:2500 overnight at 4 °C. The membrane was washed three times with tris-buffered saline solution containing Tween 20 for 10 min, and then, secondary antibodies were added at a ratio of 1:5000 for 2 h at room temperature (RT). The transferred protein band on the polyvinylidene difluoride membrane was visualized using an LAS 4000 system (GE Healthcare, Little Chalfont, UK) by inducing an enhanced chemiluminescence reaction. Antibody treatment was performed using primary antibodies, and the signal intensity was quantified using ImageJ software (NIH).

### 4.8. Confocal Microscopy 

For confocal microscopy, FL83B hepatocytes were cultured in a 3 cm plate of cover glass (Mattek Corp, Lauda-Königshofen, Baden, Germany), differentiated, and treated with a dose of EF-2001. To facilitate observation of the nucleus, cells were cultured for 10 min by immobilizing paraformaldehyde at RT with fluorescent 4′,6-diamidino-2-phenylindole diluted in PBS. To visualize triglycerol, the cells were incubated with fluorescent BODIPY 493/503 dye GFP (Thermo Fisher Scientific, Waltham, MA, USA) diluted in PBS for 30 min at RT with paraformaldehyde fixation. GFP expression was visualized using a LSM710 confocal microscope (Carl Zeiss, Oberkochen, Germany).

### 4.9. Statistical Analysis

The experimental results are expressed as mean ± standard error (SE). Analysis of variance and paired or unpaired t-tests were performed for statistical analysis, as appropriate. Statistical *p*-value < 0.05 was considered statistically significant. All experiments were performed at least three times.

## 5. Conclusions

**Figure 9:**
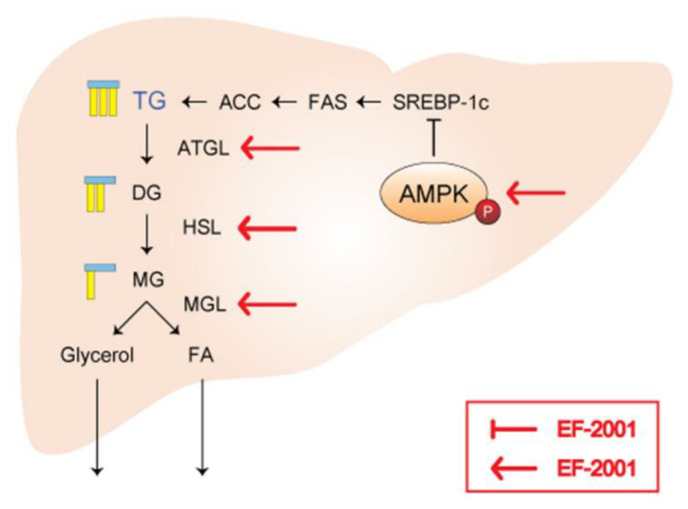
Schematic diagram of the mechanism of EF-2001 on FL83B hepatocytes and HFD-induced hepatic steatosis.

In this study, we identified the mechanism of EF-2001 in hepatic lipid accumulation in OA-induced FL83Bs. EF-2001 inhibited hepatic lipid accumulation in OA-induced FL83Bs, notably regulating lipogenesis by activating the AMPK signaling pathway in FL83B lipid accumulation. EF-2001 reduced the phosphorylation of downstream signals, such as SREBP-1C and FAS, through the SREBP-1C signaling pathway. However, EF-2001 induced the activation of lipase enzymes, such as ATGL and MGL. In addition, AMPK phosphorylation was increased by EF-2001 in AICAR and compound C treatments that targeted AMPK. Based on our results, we found that EF-2001 targeted the AMPK signaling pathway during FL83B hepatic lipid accumulation (Figure 9). We suggest that EF-2001 regulate HFD-induced abnormal hepatic lipid accumulation in the liver. Therefore, EF-2001 may prevent NAFLD associated with hepatic lipid accumulation and could potentially be used as a basis for the treatment of these diseases.

## Data Availability

The data supporting the findings of this study are available from the corresponding author upon reasonable request.
